# Geographic Range Expansion for Rat Lungworm in North America

**DOI:** 10.3201/eid2107.141980

**Published:** 2015-07

**Authors:** Emily M. York, James P. Creecy, Wayne D. Lord, William Caire

**Affiliations:** University of Central Oklahoma, Edmond, Oklahoma, USA

**Keywords:** real-time PCR, quantitative PCR, Sigmodon hispidus, hispid cotton rat, zoonoses, rat lungworm, Angiostrongylus cantonensis, Parastrongylus cantonensis, geographic range expansion, parasites, Oklahoma, Louisiana, United States

## Abstract

Using quantitative PCR analysis and DNA sequencing, we provide evidence for the presence of rat lungworm (*Angiostrongylus cantonensis*) in Oklahoma, USA, and identified a potentially novel rat host (*Sigmodon hispidus*). Our results indicate a geographic range expansion for this medically and ecologically relevant parasite in North America.

Emerging infectious diseases negatively impact humans and wildlife, causing disease outbreaks and deaths and local and global extinctions (*1*). Zoonotic disease emergence or re-emergence results from numerous factors (e.g., globalization of trade, increased interaction of humans and animals, anthropogenic climate change) that function independently or synergistically (*2**,**3*). Consequently, the means by which parasitic zoonoses are studied must be constantly advanced to promote identification, control, and prevention of outbreaks.

The rat lungworm, *Angiostrongylus* (*Parastrongylus*) *cantonensis*, causes eosinophilic meningitis in humans (*4*) and various disease manifestations (meningoencephalitis, neurologic disorders) in atypical host species, including wildlife and captive animals (*5*). Transmission of these worms occurs by ingestion of third-stage larvae in raw or undercooked intermediate or paratenic hosts (*6*). Although variable among geographic regions and within host species, the prevalence of rat lungworms might be high under favorable conditions (*7*).

The occurrence of *A. cantonensis* rat lungworms has been documented worldwide, and its distribution has been attributed largely to the spread of intermediate molluscan host species (e.g., *Achantina fulica*) and definitive rodent host species (e.g., *Rattus* spp.) (*8*). Moreover, host specificity of rat lungworms is highly plastic, which contributes to its continuous geographic expansion (*4*). These factors indicate that the rat lungworm is an emerging zoonotic pathogen of concern to humans and wildlife, and therefore provides an excellent opportunity to evaluate the sensitivity and effectiveness of epidemiologic surveying techniques.

## The Study

We evaluated the current distribution and potential spread of the rat lungworm within areas of the Gulf Coast region and midwestern United States by sampling rodent populations in regions of Louisiana and Oklahoma that were predicted by an ecologic niche model to contain suitable and unsuitable habitat (*9*). We used a quantitative PCR (qPCR) TaqMan assay (Life Technologies, Foster City, CA, USA) (*10*) to test for the parasite in tissue samples and further evaluated these samples through DNA sequencing analysis.

We trapped animals during the spring, summer, and fall months during 2010–2012. A total of 43 rodents and 3 shrews were collected from McCurtain County in southeastern Oklahoma, and 42 rodents were collected in Louisiana (Technical Appendix). We also obtained 56 *Rattus norvegicus* rat brain and lung tissue samples from the City of New Orleans Mosquito, Termite, and Rodent Control Board. Blood, lung, and brain tissue samples were collected from the rodents. Flotation was performed on all 148 lung samples, and all samples were negative for adult *A. cantonensis* rat lungworms.

Known adult rat lungworms were used as controls for molecular analyses (Technical Appendix). Cellular DNA was extracted from rodent blood and brain samples. We tested for rat lungworm internal transcribed spacer 1 (ITS1) DNA by using a TaqMan qPCR on an ABI 7500 system (*10*). A total of 134 blood samples and 137 brain samples contained DNA suitable for analysis. After qPCR, 34 of the 271 total tissue samples were classified as putatively positive for rat lungworm and sequenced, generating a 267-bp fragment of the ITS1 region (Technical Appendix).

On the basis of DNA sequencing, 3 brain samples were identified as containing *A. cantonensis* DNA (GenBank accession nos. KP231729, KP231728, and KP231727). These brain tissue samples were obtained from 3 rodents (host catalog nos. 32, 70, and 76), which were identified as 1 Hispid cotton rat (*Sigmodon hispidus*) and 2 brown rats (*Rattus norvegicus*), respectively (Table 1). A comparison of the 3 brain samples with those in GenBank by BLAST analysis (http://blast.ncbi.nlm.nih.gov/Blast.cgi) showed a match with rat lungworm (GenBank accession nos. GU587762.1 and GU587759.1) (Table 2).

**Table 1:** DNA sequences generated from 3 rat brain samples positive for *Angiostrongylus cantonensis* rat lungworms, United States*

Rat host species	Trapping location	Host catalog no.	Sequence, 5′→3′
*Sigmodon hispidus*	Red Slough WMA, OK	32	TTCATGGATGGCGAACTGATAGTATCATCGCATATCTACTATACGCATGTGACACCTGATTGACAGGAAATCTTAATGACCCAAGTATAATGTTTCAATGGGCGCCAACGTAGCAACAGAACAGTTTTTCACACGTGAAAATGTGGAACGAGATACACAGGATGtatatataTATATATATATATATACACATATATRTGTGTRTGGAAATAGATATACTAKCTTCAGMGAKGRWKCGSGYGATTCGCGTATCTAAGAAAAACACA
*Rattus norvegicus*	New Orleans, LA	70	TTCATGGATGGCGAACTGATAGTGTCATCGCATATCTACTATACGCATGTGACACCTGATTGACAGGAAATCTTAATGACCCAAGTATAATGTTTCAATGGGCGCCAACGTAGCAACAGAACAGTTTTTCTACACGTGAAAATGTGGAACGAGATACACAGGATGTATATATATATATATATACACATATATATGTGTATGGAAATTGATATACTAGCTTCAGCGATGGATCGGTCGATTCGCGTATCGATGAAAAACGCATCTA
*Rattus norvegicus*	New Orleans, LA	76	TTCATGGATGGCGAACTGATAGTATCATCGCATATCTACTATACGCATGTGACACCTGATTGACAGGAAATCTTAATGACCCAAGTATAATGTTTCAATGGGCGCCAACGTAGCAACAGAACAGTTTTTCTACACGTGAAAATGTGGAACGAGATACACAGGATGTATATATATATATATATACACATATATATGTGTATGGAAATTGATATACTAGCTTCAGCGATGGATCGGTCGATTCGCGTATCGATGAAAAACGCAGCTA

*WMA, wildlife management area.

**Table 2:** BLAST* results for sequences from 3 rat brain samples, United States†

Host catalog no.	Rat host species	Trapping location	Match, %	Coverage, %	e value
32	*Sigmodon hispidus*	Red Slough WMA, OK	92	98	5 × 10^–105^
70	*Rattus norvegicus*	New Orleans, LA	99	100	3 × 10^–130^
76	*R. norvegicus*	New Orleans, LA	99	100	1 × 10^–133^

*http://blast.ncbi.nlm.nih.gov/Blast.cgi †WMA, wildlife management area.

All sequences were aligned by using MUSCLE in MEGA 5.2 (http://www.megasoftware.net/), manually inspected for consensus, and compared with the 267-bp fragment generated from the known sample of rat lungworm. Maximum-likelihood phylogenetic analysis was performed by using sequence data for the ITS1 region of *A. cantonensis* (GenBank accession no. GU587759.1), 2 closely related species, *A. vasorum* (the French heartworm, GU733324.1) and *A. costaricensis* (a parasitic nematode, (GU587745.1), and sequences obtained from the rodent hosts. Maximum-likelihood phylogenetic analysis grouped sequences from rat hosts 32, 70, and 76 with *A. cantonensis* with high bootstrap support (Figure).

**Figure:**
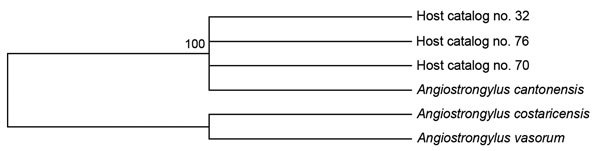
Maximum-likelihood bootstrap consensus phylogenetic tree showing the relationship between rat lungworm sequences generated in this study and other *Angiostrongylus* spp., United States. Tree was generated by using a Tamura 3-parameter model. Value along the branch is a bootstrap value.

## Conclusions

Because the rat lungworm poses a major health risk to humans and wildlife worldwide, more work is needed to shed light on the location, dispersal, and influence of this parasite in new geographic regions. Although previous reports document rat lungworms in the Gulf Coast region of the United States (*5**,**11*), little is understood regarding their prevalence within definitive hosts and their dispersal throughout the southeastern United States. As expected, our analysis indicated that *R. norvegicus* rats from Louisiana harbored rat lungworms. Positive samples were collected from densely populated areas with high tourist activity, thereby increasing the risk for transmission to humans. Moreover, rat lungworms were identified outside their known habitat and in a new rat host species (*S. hispidus*) in Oklahoma, an area predicted to lack suitable habitat for the parasite (*9*). Our results provide a new perspective on the distribution of rat lungworms in the United States and indicate a northward range expansion that substantially increases the risk for disease spread within humans and wildlife.

Because endemic and novel pathogens require different and highly specialized disease management strategies, it is crucial to determine whether a pathogen is novel or endemic (*12*). Previous work has described the *A. cantonensis* rat lungworm as a novel pathogen in the southeastern United States. However, it is now characterized as endemic to this region, and our results strongly support this notion (*11*). Such changes in epidemiologic classification of rat lungworms accentuate the need for techniques that monitor the extent to which parasites infiltrate new geographic areas and potentially pose threats to humans and native wildlife. One such threat includes an increasing prevalence of angiostrongyliasis, which should receive increased scrutiny in patients with eosinophilic meningitis from localities characterized by paratenic and intermediate hosts.

Rat lungworm was found in a previously undocumented mammalian host, *S. hispidus* rats, which strongly suggests that this parasite is an endemic pathogen. Although vegetation is their primary food source, *S. hispidus* rats will eat invertebrates (*13*). Whether these rats directly (by intentional consumption of host) or indirectly (by consumption of host or free third-stage larvae on vegetation) consume the parasite, we cannot rule out the possibility that acquisition of the parasite could occur in this species and enable further range expansion for rat lungworms. *S. hispidus* rats are a known host for another closely related *Angiostrongylus* species, *A. costaricensis*, which lends additional support for the notion that *S. hispidus* rats might act as a host for rat lungworms. Alternatively, *S. hispidus* rats might simply be an accidental/dead end host for this parasite. Although wildlife might become infected with the parasite, not all wildlife are definitive hosts (*5**,**11*). Additional field and laboratory studies will clarify the role that *S. hispidus* rats play in the spread of the rat lungworm.

Because many terrestrial species remain taxonomically nondescribed, there is strong potential for continual emergence of unknown pathogens worldwide (*14*). Global travel, human encroachment into wildlife habitat, and climate change will influence distribution and emergence of disease (*2**,**15*). By incorporating field epidemiology with molecular genetic techniques to determine the geographic distribution of pathogens, major advances can be made in preventing the spread of wildlife diseases to human populations. Our results illustrate this point and highlight the need for future work to incorporate and refine these techniques and their application to epidemiology and wildlife disease surveillance.

**Technical Appendix.** Additional details on geographic range expansion for rat lungworm in North America.
