# Supplementary material for: Geographic Range Expansion for Rat Lungworm in North America
Source: Emerg Infect Dis. 2015 Jul;21(7):1234–6. doi: 10.3201/eid2107.141980 (PMC4480392; doi:10.3201/eid2107.141980)
Supplement: Supplementary file 1 — Technical Appendix. Additional details on geographic range expansion for rat lungworm in North America. [file 14-1980-Techapp-s1.pdf]

Article DOI: http://dx.doi.org/10.3201/eid2107.141980

Geographic Range Expansion for Rat Lungworm in North America

Technical Appendix

Technical Appendix Table 1. Location, species, and number of mammals collected from each area that were tested for *Angiostrongylus cantonensis* rat lungworms, United States

Trapping location	Species	Common name	No. caught
McCurtain County, OK	*Neotoma floridana*	Eastern wood rat	2
McCurtain County, OK	*Blarina carolinensis*	Southern short-tailed shrew	3
McCurtain County, OK	*Oryzomys palustris*	Marsh rice rat	2
McCurtain County, OK	*Peromyscus leucopus*	White-footed mouse	1
McCurtain County, OK	*Reithrodontomys fulvescens*	Fulvous harvest mouse	7
McCurtain County, OK	*Sigmodon hispidus*	Hispid cotton rat	30
New Orleans Parish, LA	*Rattus norvegicus*	Brown rat	36
East Baton Rouge Parish, LA	*Rattus*	Black rat	3
Calcasieu Parish, LA	*Rattus norvegicus*	Brown rat	8

Technical Appendix Table 2. DNA sequence fragments of the ITS1 region generated from a known *Angiostrongylus cantonensis* rat lungworm specimen, United States *

Region	Sequence, 5′→3′
Species-specific region, 105 bp	TTTCATGGATGGCGAACTGATAGTATCATCGCATATATACTATACGCATGTGACACCTGAT TGACAGGAAATCTTAATGACCCAAGTATAATGTTTCAATGGGCGC
267-bp fragment used in phylogenetic analysis	TTCATGGATGGCGAACTGATAGTATCATCGCATATATACTATACGCATGTGACACCTGATT GACAGGAAATCTTAATGACCCAAGTATAATGTTTCAATGGGCGCCAACGTAGCAACAGAA CAGTTTTTCTACACGTGAAAATGTGGAACGAGATACACAGGATGTATATATATATATATAC ACATATATATATGTGTATGGAAATTGATATACTAGCTTCAGCGATGGATCGGTCGATTCGC GTATCGATGAAAAACGCATCTAAA

*ITS1, internal transcribed spacer 1.

Technical Appendix Table 3. Presence of *Angiostrongylus cantonensis* DNA in tissue types from host species, United States

Host species	Common name	Tissue type	No. samples positive/no. samples screened
Rattus norvegicus	Brown rat	Brain	2/97
		Blood	0/98
		Lung	0/100
Rattus	Black rat	Brain	0/3
		Blood	0/3
		Lung	0/3
Sigmodon hispidus	Hispid cotton rat	Brain	1/27
		Blood	0/26
		Lung	0/30
Neotoma floridana	Eastern wood rat	Brain	0/2
		Blood	0/2
		Lung	0/2
Oryzomys palustris	Marsh rice rat	Brain	0
		Blood	0
		Lung	0/2
Peromyscus leucopus	White-footed mouse	Brain	0/1
		Blood	0/1
		Lung	0/1
Reithrodontomys fulvescens	Fulvous harvest mouse	Brain	0/7
		Blood	0/4
		Lung	0/7

Host species	Common name	Tissue type	No. samples positive/no. samples screened
Blarina carolinensis	Southern short-tailed shrew	Brain	0
		Blood	0
		Lung	0/3
